# Advances in Dealloying of Ti and Ti-Based Alloys for Biomedical Applications

**DOI:** 10.3390/ma18184424

**Published:** 2025-09-22

**Authors:** Kirti Tiwari, Deepti Raj, Paola Rizzi, Federico Scaglione

**Affiliations:** Dipartimento di Chimica e Centro Interdipartimentale NIS (Nanostructured Surfaces and Interfaces), Università di Torino, Via Pietro Giuria 7, 10125 Turin, Italy; tiwari.kirti1998@gmail.com (K.T.); deeptiraj947@gmail.com (D.R.); federico.scaglione@unito.it (F.S.)

**Keywords:** dealloying, Ti and its alloys, bulk metallic glass, crystalline alloy, shape memory alloy, biomaterials

## Abstract

Dealloying technique has been used for centuries as an attractive method for producing porous surfaces by removing one or more undesirable elements from the surface. Since early 2000s, the technique has been further developed for understanding the dealloying mechanism and tailoring it to produce chemically homogeneous materials with nanoporous (np) morphology. Dealloying has found numerous applications such as sensors, catalysts, as well as in the biomedical field, which is fairly recent and has attracted great attention on this topic. This review investigates the dealloying technique for preparing nanoporous materials and nanoporous surfaces by using different modification routes on various types of Ti-based alloys for biomedical implant application. There has been significant growth in studying dealloying of crystalline, amorphous, shape memory, and composites-based Ti alloys. This review aims to summarise the findings from literature and discuss the scope of this technique and challenges involved for future aspects.

## 1. Introduction

Dealloying is an ancient technique used by ironsmiths and jewellers for removing less noble elements from the surface of metallic materials like copper alloys containing gold and silver (a possible example is the tumbaga alloy, a natural copper reach alloy) in order to make them appear as pure noble metals like gold. An increasing surge in scientific study and application of dealloying has been observed in the 20–21st century [1,2]. Originally regarded as a corrosion phenomenon in the case of brass leading to dezincification, this technique is now recognized as a simple and effective method involving the selective dissolution of less noble atoms from a solid solution or an amorphous alloy. The remaining, more noble atoms then undergo self-organization through surface diffusion, resulting in a highly porous material with a large surface area and a widespread pore-ligament framework. It is worth noting that the dealloying process can involve the whole volume of the material, starting from the surface toward the bulk, enabling the production of nanoporous (np) metals of different shapes [3,4]. There has been significant contribution, in the last two decades, by researchers in unveiling the dealloying mechanism and the porosity evolution, developing working model to understand fundamentals of dealloying and new techniques to create np materials with high porosity and surface area [5]. It has attracted new interests in applications for sensors, catalysts, energy storage/conversion, automobile, filtration of metal ions, organic dyes, microbial organisms [6]. Moreover, there is an interest to focus on its application in biomedical field for the development of efficient implant materials, which are important for replacing damaged and diseased parts of body for improving the quality of a patient’s life [7]. Tailoring the surface chemistry and morphology of these materials is vital in order to promote biocompatibility and prevent biofilm formation which commonly causes failure of implants. Other reasons behind implant failure include release of toxic elements in the body, debris generation, corrosion, lower strength for load bearing application, and biological response due to the presence of foreign material. The failure of implants leads to the requirement of a revision surgery which can be painful as well as expensive for the patients while holding a fairly lower success rate compared to the first surgery [8,9,10]. The chances of success of an implant highly depend on good osseointegration and antibacterial properties [11,12]. Therefore, it is necessary to modify the surface morphology and chemical composition of the implant materials for fabricating a biocompatible surface possessing antibacterial, antibiofouling and antibiofilm properties. If biocompatibility and antibacterial activity is considered, it is worth noting that in most cases it is vital to operate morphological and chemical changes on the surface of the samples, i.e., on the material that is in contact with the body, leaving all the original bulk properties of the material unaltered. For this reason, this study is mainly focused on the surface modification produced by the dealloying processes. Titanium based biomaterials, credited to their superior mechanical properties, longevity and biocompatibility, have been extensively used over decades for biomedical implants like stents, heart valves, hip and knee prostheses, dental screws, bone screws, nails and blood contacting devices [13,14,15,16,17,18,19]. Although various techniques have been employed for developing capable implant surfaces such as blasting, acid etching, anodization, plasma spraying and hydrothermal treatment, dealloying remains comparatively less explored for production of porous surfaces on Ti and its alloys [20,21,22,23].

**Figure 1:**
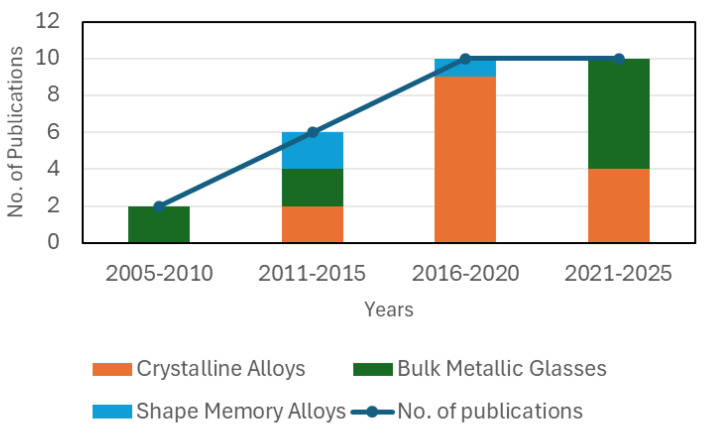
Trends in producing np-titanium by dealloying method, since 2005 to 2025, summarising the number of publications with research focus on the class of materials.

This review paper aims to highlight the studies focussed on fabrication of Ti and its alloys via dealloying in order to discuss their potential with regard to the development of porous structures for biomedical implants. In Figure 1 we have summarised the relevant scientific works done by researchers since early 2000s to 2025 emphasising the increasing focus of researchers towards the improvement of the dealloying technique for application in biomedical field. The calculation used for producing Figure 1 was merely done by counting the number of articles collected for the study and dividing them by the year of publication. The focus has been towards crystalline alloys primarily, while other class of materials like bulk metallic glasses and shape memory alloys are being explored. As this research field is gaining momentum, it is necessary to give a direction to it underlining the advances in surface science.

## 2. Types of Dealloying Techniques

**Figure 2:**
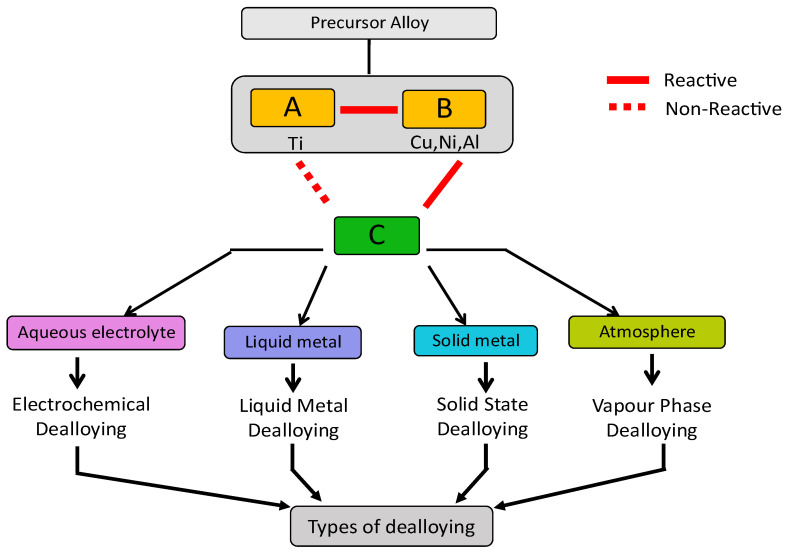
Schematic representation of dealloying techniques.

The schematic representation shown in Figure 2 summarises the different dealloying techniques. The precursor alloy here is a solid solution formed with two elements, A and B, where B needs to be selectively removed. The choice of dealloying media or electrolyte, denoted here as C, depends on their chemical reactivity towards B. Here, the element C is reactive towards B but inert towards A. This leads to the targeted dissolution of B into C from the A-B precursor alloy, leaving behind A as a nanoporous material. A number of different dealloying techniques have been studied by researchers since 2000s to explore new options for dealloying media beyond aqueous electrolytes.

Dealloying can be classified in various ways depending on the medium used. When the dissolving medium is an aqueous solution of acids or bases, the process is referred to as chemical or electrochemical dealloying. In this case, the chemical behavior of the alloy and its interaction with the liquid medium determines the removal of the targeted element. Another technique of dealloying involves the use of a liquid metal, known as liquid metal dealloying. In this process, the molten metal, chosen for its specific chemical and physical properties, facilitates the separation of the B element from the alloy. Solid-state dealloying occurs when the medium employed is a solid metal. In this scenario, the B element is removed through interaction with the solid material. Finally, Vapor Phase Dealloying is a process that employs high temperatures and low pressures to remove one element from the alloy thanks to its evaporation, living behind a porous material. In general, this classification reflects the various technological and physicochemical approaches employed to achieve dealloying based on the characteristics of the medium and operating conditions. To allow an easier comparison among the different strategies used, in Table 1 a summary of the principal characteristics is reported. Further details on these dealloying techniques are presented below.

### 2.1. Chemical and Electrochemical Dealloying

**Figure 3:**
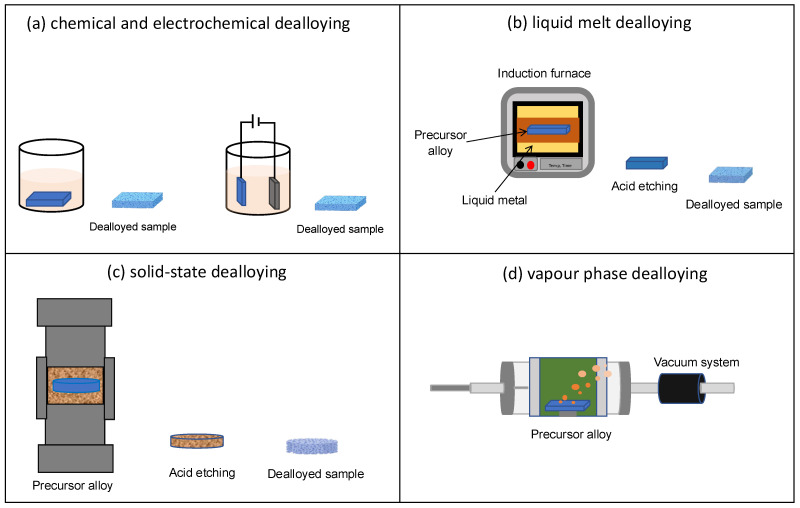
Schematic representation of (**a**) chemical and electrochemical dealloying (**b**) liquid melt dealloying technique (**c**) solid-state dealloying technique (**d**) vapour phase dealloying technique.

Chemical dealloying involves free immersion of precursor alloy in the etching solution as shown in Figure 3a. It is a spontaneous process which can be performed at room temperature or at elevated temperatures and offers various advantages such as simplicity, affordability and the possibility of easy scale-up for commercialization [24,25]. The morphology of the dealloyed sample can be controlled and optimized by regulating the experimental conditions like concentration and pH (acid/base) of the electrolyte, treatment time and temperature. However, this method is not suitable for alloys with elements having similar chemical properties as it becomes difficult to ensure selective etching [26].

Electrochemical dealloying is another widely used technique to selectively remove one or more components from a metallic alloy (Figure 3a). The process can be carried out in potentiostatic or galvanostatic mode, applying a constant electric potential or a constant current to the alloy, which acts as the working electrode in an electrochemical cell. The cell also includes a reference electrode, a counter electrode, and an aqueous solution containing acids or bases selected according to the alloy composition [27]. These techniques have been widely studied on Au-Ag based alloys, where Ag is less noble than Au and it is selectively dissolved, producing np-Au [28]. The experimental parameters can be optimized by adjusting parameters such as the electrolyte concentration, temperature, alloy composition and applied current or potential to tailor the morphology of the sample. This method is also facile and inexpensive but, obviously, precursor alloys containing elements with similar electrode potential values cannot be dealloyed using this method [6,26]. Some examples of nanoporous metals produced by dealloying using this technique are Pt, Pd, Ag, Cu, Ni, Al [29,30,31,32,33,34,35,36,37].

### 2.2. Liquid Metal Dealloying

Liquid metal dealloying (LMD) technique uses a metallic melt for dealloying a sample. This method is based on the difference in enthalpy of mixing of elements present in precursor alloy with respect to the liquid melt. The elements selected as liquid melts are based on the theory that at certain dealloying temperature, when the solid precursor alloy is immersed in the liquid melt, the constituent element that has negative enthalpy of mixing with the metallic melt gets diffused while that with positive enthalpy of mixing remains on the surface of the alloy. The atoms present on the surface of the sample rearrange and form ligaments and pores via surface diffusion [38,39]. The schematic representation of liquid melt dealloying is shown in Figure 3b. For better understanding, Ti-Cu alloys can be considered. When a Ti-Cu alloy is immersed in a Mg molten bath, Cu diffuses in the melt, being miscible in Mg, and, upon colling, forms Mg_2_Cu phase. Contrary, Ti is immiscible in liquid Mg and it remains solid with adatoms moving by surface diffusion producing ligaments and pores in which the molten Mg can penetrate enabling the dealloying of the whole sample volume. At the end of the dealloying process the sample is cooled at room temperature and it is immersed in HNO_3_ to selectively etch Mg_2_Cu phase producing np Ti-rich material. This technique provides new possibilities for the production of different nanoporous metals, through the selection of precursor alloys and suitable elements for liquid melt. This phenomenon was first explained by Harrison and Wagner in 1959 [40] and then re-addressed by Wada et al., in 2011 [41,42]. This technique can be useful for dealloying less noble elements like Ti, which is prone to oxidation, form a passive oxide film and have positive reduction potential. For all these reasons, nanoporous Ti is challenging to be produced by using chemical and electrochemical dealloying routes [43]. However, for the liquid metal dealloying there are some limitations like maintaining proper working conditions which requires high temperature for preparing metallic melt that involves high cost of operation [26]. This technique has been widely used to fabricate nanoporous materials like Ti, Nb, V, Fe, Cr, Mn, Ta, Cu [44,45,46,47].

### 2.3. Solid-State Dealloying

Solid-state dealloying (SSD) is also referred to as solid metal dealloying (SMD). In this technique, which is similar to LMD in thermodynamic aspects, one element is selectively removed via solid-state diffusion mechanism into a solid metal solvent to produce a bi-continuous nanoporous structure as shown in Figure 3c. Unlike LMD, where heat of enthalpy and relative miscibility of elements is an important criterion, SMD is, thus, dependent on the interdiffusion kinetics of the metal solvent with the components of the alloy. Slower transport kinetics and difference in the diffusion coefficients of the solid metals play a vital role for a successful dealloying. The interdiffusion kinetics of metal solvent should be slower with at least one order of magnitude compared to other components of the alloy. Dealloying occurs at the metal-solvent interface and depending on the rate of diffusion and heat of mixing of the concerned element in the metal solvent, the reorganization of atoms at the interface is determined. This method requires processing temperatures below the melting point of the metal solvent and it promotes the formation of finer structures with respect to the previous described dealloying processes [48]. It is also cost-effective technique which provides a wide window for varying processing time and temperature and enabling more control over the volume fraction of the components present in the alloy. This is an important criterion as it influences the morphology of the porous networks [49]. SSD was first reported by Wada et al., in 2016 [50] where the authors proposed a new strategy for producing porous structures developed on (Fe_0.8_Cr_0.2_)_50_Ni_50_ alloy. It involved atomic diffusion of Ni in Mg occurring at a temperature lower than the melting point of the Mg powder which was used as the dealloying medium. This treatment resulted in the formation of a nanoporous material with very fine morphology rich in Fe-Cr phases and lower Ni content, composition being Fe_76.3_Cr_20.4_Ni_3.3_. Ni_2_Mg phase formed due to solid-state diffusion was later etched using HNO_3_ solution. Currently, thin film substrates are used for studying SSD mechanism. McCue et al., in 2017 [51], developed dense metal composites by solid-state dealloying of a binary precursor alloy deposited on solid metal solvent made of Cu and Zr. This study suggested the use of SSD in creating multi-layered films with different compositions of precursor alloy and metal solvent which can be dealloyed simultaneously. The authors studied requirements and kinetics for a solid-state dealloying by using thin film of Ni_55_Fe_45_ (750 nm thickness) and Ti_65_Ta_35_ (700–800 nm thickness) alloys deposited on Cu and Zr substrates respectively. In this design Cu and Zr remain immiscible with Ta and Fe but miscible with Ti and Ni. The dealloying occurred by diffusing the miscible components from the deposited thin film toward the substrate. The interdiffusion kinetics were studied and the authors proposed Ti-Ta/Zr as the ideal model for further investigations like finding critical composition for SSD to occur and mechanism of morphology formation. The influence of volume variation in metal solvent and the substrate, considering their diffusion kinetics, was also investigated as it is an important parameter. It was concluded that, altering the composition of parent alloy interpenetrating, metal nanocomposite can be produced where volume fraction of each phase gradually changes upon dealloying. Additionally, it is possible to dealloy thin films with multiple layers of metal solvent which can then be simultaneously dealloyed through the entire layers thus, eradicating the limitations of performing SSD with only one substrate. Recently in 2019, Yao Shi et al. [52] fabricated ultrafine ligaments of nanoporous Ti. Samples were prepared by ball milling Ti-Cu powders with Mg powder followed by heat treatment to form a phase of Mg-Cu, the samples were then chemically etched in 1 M HNO_3_ solution to remove Mg-Cu phase and produce nanoporous Ti.

### 2.4. Vapour Phase Dealloying

Vapour phase dealloying (VPD) is a technique involving evaporation of one or more elements from the precursor alloy to fabricate different morphologies as shown in Figure 3d. This technique uses the difference in the melting and boiling point of the elements present in the alloy as well as the difference in their saturated vapour pressure. The dealloying temperature is maintained below the melting point of the precursor alloy in accordance with the Kirkendall effect [53,54]. This method is quite facile and environment friendly However, this type of treatment was till now limited to Zn based alloy systems for dealloying elements like Ni and/or Co [55,56]. This phenomenon often attributed as Kirkendall effect was observed by Ballufi and Alexander back in 1952 [57,58] where the authors reported dezincification of brass in vacuum, which led to the formation of close porosities instead of interdiffusion surface rearrangement which produces bicontinuous open porosity. The authors studied diffusional changes in Ag from vapour phase into a thin gold wire at 1213 K in normal and parallel direction of diffusion. Recently this technique received attention when Zhen Lu et al. [59] developed a prototype of Co_5_Zn_21_ alloy by mechanically milling powders at room temperature and melt spinning to produce thin ribbons. The ribbons were heated in an inert atmosphere at 773 K and 100 Pa for 5 to 120 min and the resultant dealloyed samples were found to be rich in Co. Additionally Yujun Shi et al. [60] synthesized bulk nanoporous Co with face cantered cubic structure from a Co_5_Zn_21_ alloy foils. The nanoporous Co had a surface area of 1.06 m^2^g^−1^ and large pore size distribution with average pore diameter around 400 nm and ligament size 0.36 ± 0.07 µm. Jiuhui Han et al. [56] fabricated 3D bicontinuous nanoporous Ni and Ge from Ni-Zn and Ge-Zn precursor alloys with tunable pore size by varying the temperature, time of treatment. VPD has been attempted for dealloying Mn-Zn, Ni-Zn and Al-Zn alloys for fabricating nanoporous Mn, Ni and Al [61,62,63].

This technique is being utilized on various materials as it allows the tunability of pore size from micron to nano scale by controlling the dealloying parameters like temperature, time and pressure. Since it does not involve any chemical or electrochemical route of fabrication, the method is versatile and it allows its application on various alloys without the need of considering their electrical conductivity or chemical stability. Importantly, it is an economical, pollution-free technique and affordable where recovery of the evaporated elements such as zinc in the vacuum system is possible. Thus, this method holds great potential.

## 3. Fabrication of Nanoporous Ti and Its Alloys

Nanoporous Ti (np-Ti) surface shows biocompatibility due to presence of an inert passivated oxide layer with a morphology and roughness appropriate for human tissues to adhere and proliferate. It can be fabricated via the dealloying techniques previously discussed in Section 2. Various factors need to be considered such as the alloy composition, type of etching electrolyte, etching time and temperature and the applied potential in case of electrochemical dealloying. Researchers have thoroughly studied the advantages of developing np-Ti structures for enhanced cell adhesion, hemocompatibility and antibacterial properties. Studies have also shown that np-surfaces coated with bioactive polymers can boost cell adhesion, proliferation and differentiation and reduce platelet adhesion and thrombus formation based on the site of application [64,65,66,67]. Other studies have shown that drug loading into the np-structures can alter the drug release kinetics for a prolonged biocompatibility [68,69]. In the coming section the dealloying of titanium-based alloys is discussed with respect to the method and type of parent alloy used for the synthesis of the np-structure, i.e., crystalline, amorphous, composites and shape memory alloy.

### 3.1. Ti-Based Crystalline Alloys

**Figure 4:**
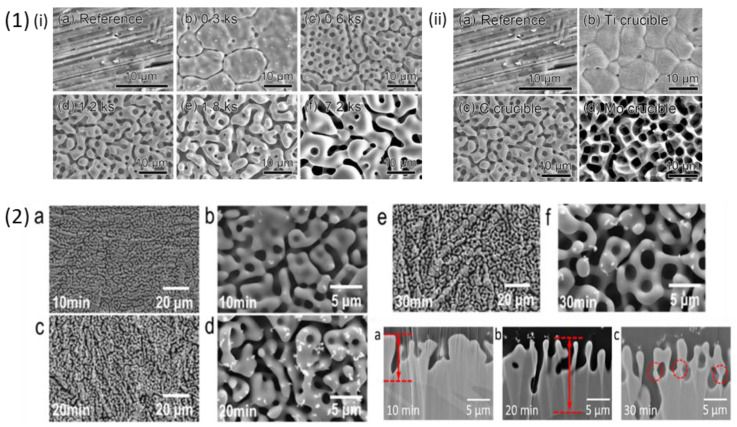
(**1**) [78] (**i**) SEM images of Ti6Al4V untreated and dealloyed samples in Mg melt at 1148 K for (**a**) untreated (reference) sample, (**b**) 0.3 ks, (**c**) 0.6 ks, (**d**) 1.2 ks, (**e**) 1.8 ks and (**f**) 7.2 ks. (**ii**) Dealloyed sample at 1148 K for 1.2 ks in (**a**) untreated (reference) sample, (**b**) Ti, (**c**) C, and (**d**) Mo crucibles. (**2**) [79] SEM images of Ti6Al7Nb samples immersed in Mg melt at 1150 K for (**a**,**b**) 10 min, (**c**,**d**) 20 min and (**e**,**f**) 30 min followed by acid etching to remove Mg-Cu phase. (**2**) in lower right corner, SEM of cross-section of the dealloyed samples for (**a**) 10 min, (**b**) 20 min and (**c**) 30 min.

Titanium and Titanium based crystalline alloys have been used in biomedical applications since 1900s due to their various advantages like superior mechanical properties, corrosion resistance, inert nature and biocompatibility [70]. Commercially, pure Ti metal (Cp-Ti, both grade 1 and grade 2) and Ti6Al4V alloys are the most commonly used materials in the biomedical industry mostly used as components for joint replacement due to their mechanical properties [71]. Cp-Ti grade 1 and grade 2, Ti6Al4V and Ti6Al7Nb have been used for dental implant application due to presence of stable TiO_2_ oxide layer and high corrosion resistance property [72,73,74,75]. However, Ti6Al4V and Ti6Al7Nb have some limitations due to the presence of toxic elements, i.e., Vanadium (V) and Aluminium (Al) which in excess concentration can be detrimental to human body [76,77]. To avoid their release in the body environment, an attempt to dealloy Ti6Al4V alloy was performed by Yuichi Fukuzumi et al. [78] in which they immersed the alloy in Mg melt to remove the toxic elements present on the sample surface in order to enhance its biocompatibility. The choice of magnesium melt was made by considering its enthalpy of mixing with Al, Ti and V. The coarsening of the surface was studied by increasing the treatment time from 0.3 to 7.2 ks and temperature of the Mg melt from 1048 K to 1148 K. The authors observed coarser ligaments on samples treated at 1148 K for 7.2 ks in the SEM images as shown in Figure 4 (1) (i). The increase in the immersion time showed formation of coarser porous structures suggesting dependence of alloys chemical composition and surface morphology on the treatment parameters like time and temperature. The authors also studied the effect of using different types of crucibles, made of Ti, Mo and C, on dealloying. The authors then concluded that crucible materials are quite important for determining surface features like morphology and ion release of the dealloyed samples. The SEM images in Figure 4 (1) (ii) of the dealloyed samples in Mo and C crucible at 1148 K for 7.2 ks showed formation of porous structure whereas samples immersed in Ti crucible showed appearance of small, isolated pores on the grain boundaries of the sample surface which was suggested to occur due to dissolution of Ti atoms from crucible and simultaneous deposition on the sample surface during the dealloying process. The dealloyed sample was analysed using energy dispersive X-ray spectroscopy (EDX), the concentration of Ti was reported to be 88.9 wt % whereas in the untreated sample it was 90.9 wt %. The authors reported a decrease in Ti content when C crucible was used, thus change in the mass of Ti was studied by immersing Cp-Ti sample in C crucible at 1184 K for 1.8 ks and measuring its weight before and after the treatment. The mass loss of the Cp-Ti sample was measured to be around 6 mg after immersion in Mg melt which indicates a clear decline in the content of Ti. Despite the crucible types the Al and V content was reduced after the dealloying treatment. Furthermore, the dealloyed samples were immersed in simulated body fluid (SBF) for 2 weeks to study their ion release activity. The result of this study is discussed later in detail in Section 4. Another Ti6Al7Nb crystalline alloy was dealloyed by I. V. Okulov et al. using liquid melt [79]. The authors investigated the dependence of treatment parameters like time and temperature of the liquid melt for dealloying of Ti6Al7Nb alloy containing two phases, α-Ti and β-Ti. The Ti6Al7Nb samples were prepared by a sintering process and then dealloyed in Mg metallic melt for 10, 20 and 30 min to selectively dissolve Al from the sample surface. After the dealloying process Mg rich phases containing Al were formed on the sample surface which was then etched by treating in 3 M HNO_3_ solution for 30 min. The concentration of Al was reduced by 48% from the parent alloy after 30 min of immersion in Mg melt at 1150 K. The non-dealloyed and dealloyed samples were characterized using XRD, the former showed presence of dual phase of α-Ti and β-Ti whereas after dealloying the porous layer consisted mostly of β-Ti phase with reduced α-Ti content. The morphology of the dealloyed sample was studied using SEM, as shown in Figure 4 (2). The surface showed bimodal porous structures with ligament thickness of 16.1 µm and 6.8 µm, rich in Nb and lower content of Al on the surface after 30 min of dealloying. It was concluded that the thickness of the dealloyed layer increased with increasing the dealloying time. The cytotoxicity test and cell studies on the dealloyed sample are discussed later in Section 4.

**Figure 5:**
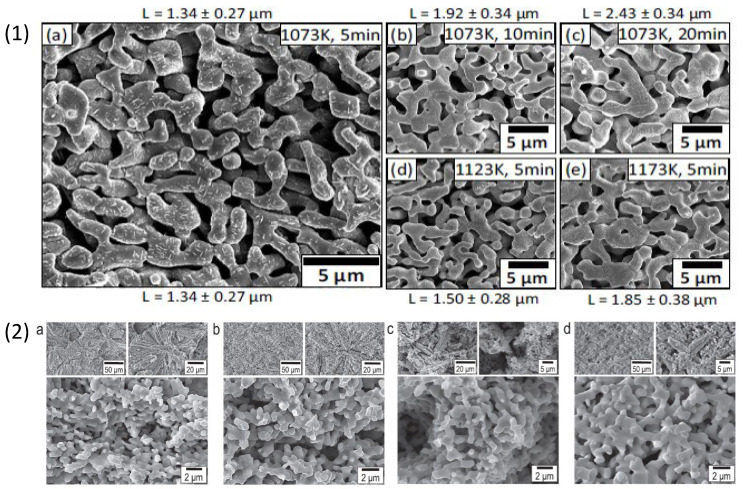
(**1**) [82] SEM image of as-dealloyed samples with ligament size (L) dealloyed at (**a**) 1073 K for 5 min (**b**) 1073 K for 10 min (**c**) 1073 K for 20 min (**d**) 1123 K for 5 min (**e**) 1173 K for 5 min. (**2**) [83] SEM images of (**a**,**b**) porous TiNb alloy and (**c**,**d**) TiFe alloy produced by dealloying. The images (**a**,**c**) were treated at 1073 K for 10 min and (**b**,**d**) 1173 K for 5 min.

There has been extensive research done on Ti-Zr based alloys due to their superior strength, biocompatibility, and corrosion resistance for application as biomedical implant material [80,81]. I. V. Okulov et al. [82] performed a comparison study between Ti6Al4V alloy and porous Ti-Zr based alloy for load bearing implant application. Porous Ti-Zr was synthesised using liquid melt dealloying technique in Mg melt. The alloy composition was selected based on values of enthalpy of mixing between Mg and elements in the alloy. The selected alloys were Ti_x_Zr_(100-x)y_Cu_100-y_ with compositions Ti_15_Zr_15_Cu_70_, Ti_20_Zr_20_Cu_60_, Ti_25_Zr_25_Cu_50_ and Ti_30_Zr_30_Cu_40_ (at%) prepared into 1 mm diameter rods using arc-melting technique. Different compositions were used to understand the parting limit of Cu, i.e., the minimum concentration at which dealloying occurs. The samples of 1.7 mm length were heated for different durations and temperatures with 130 mg of Mg enclosed in a graphite crucible in an inert atmosphere. All the samples were dealloyed in Mg bath for 5–20 min at 1073–1173 K to form a Mg-Cu phase on the surface of the alloys. The dealloyed samples were then etched in 3 M HNO_3_ for 5 h to remove Mg-rich phase. All precursor alloys showed different ligament sizes upon the dealloying treatment which were optimised by altering dealloying conditions like time and temperature. The SEM images in Figure 5 (1) show a summary of the processing parameters like duration and temperature of dealloyed Ti_15_Zr_15_Cu_70_ alloy, which contains smallest ligament size (L) of 1.34 ± 0.27 µm and lowest Young’s modulus of 3.2 ± 0.2 GPa after dealloying at 1073 K for 5 min. It was observed that increasing the time of dealloying at constant temperature led to formation of bigger ligaments, as shown in Figure 5 (1) (a–c). A similar effect on the ligament size was observed in Figure 5 (1) (a,d,e) at fixed duration with increased temperatures. Moreover, the authors observed that changing the content of Cu in the precursor alloy from 70 to 40 at % showed different shrinkage behaviour wherein the dealloyed sample showed increased solid fraction until 79 vol %. Thus, chemical composition of the precursor alloy can determine the ligament size and solid fraction of the dealloyed surface, providing an opportunity of tuning the dealloyed morphology by controlling the dealloying conditions and modifying the precursor alloy composition. The result of this study suggests the interdependency of the dealloying parameters and alloy composition on the formation of porous morphology. The dealloyed samples were evaluated for cytocompatibility using human umbilical cord perivascular cells on porous samples using Ti6Al4V alloy as control. The result from this study is discussed in Section 4 later. In another study by I. V. Okulov et al., [83] the authors altered microstructures and the mechanical properties of crystalline alloy Ti_27.2_Nb_3_Cu_69.8_ and Ti_29.2_Fe_3.9_Cu_66.9_ at % by optimising the parameters for dealloying and further coating with polymer to improve the yield strength of the dealloyed sample. Firstly, Ti_27.2_Nb_3_Cu_69.8_ and Ti_29.2_Fe_3.9_Cu_66.9_ disc was immersed in Mg melt at 1073 K for 10 min and 1173 K for 5 min in a carbon crucible. This ensured diffusion of Cu in the molten Mg leading to its selective dissolution from the sample surface. The dealloying treatment was followed by etching of Mg rich phase from the dealloyed sample surface using HNO_3_ solution for 5 h to produce np-structure. Minor additions of Fe and Nb were done for microstructural refinement as they are beta stabilizing elements. The dealloyed samples of Ti_89.4_Nb_10.6_ consist of orthorhombic α-martensite and β-Ti while Ti_88.2_Fe_11.8_ consists of only β-Ti phase. The porous samples were coated with liquid bisphenol F epoxy (BPF) resin polymer. The yield strength of the TiFe composite samples increased from 89 ± 10 MPa to 260 ± 15 MPa and of TiNb composite from 72 ± 6 MPa to 205 ± 15 MPa, due to impregnation of polymer into the pores of the scaffold which led to increase in the mechanical performance. The morphology of dealloyed samples, as presented in SEM images in Figure 5 (2) (a–d), showed bicontinuous network of ligaments, sized 0.55 ± 0.11 µm at 1073 K and 0.75 ± 0.17 µm at 1173 K for Ti_89.4_Nb_10.6_ at% and Ti_88.2_Fe_11.8_ at % respectively. The morphology of the samples consists of micrometre size dendrites embedded in a porous matrix of the samples. The Young’s modulus of the two composites was near 5 GPa which suggests promising application for biomedical implants [84].

**Figure 6:**
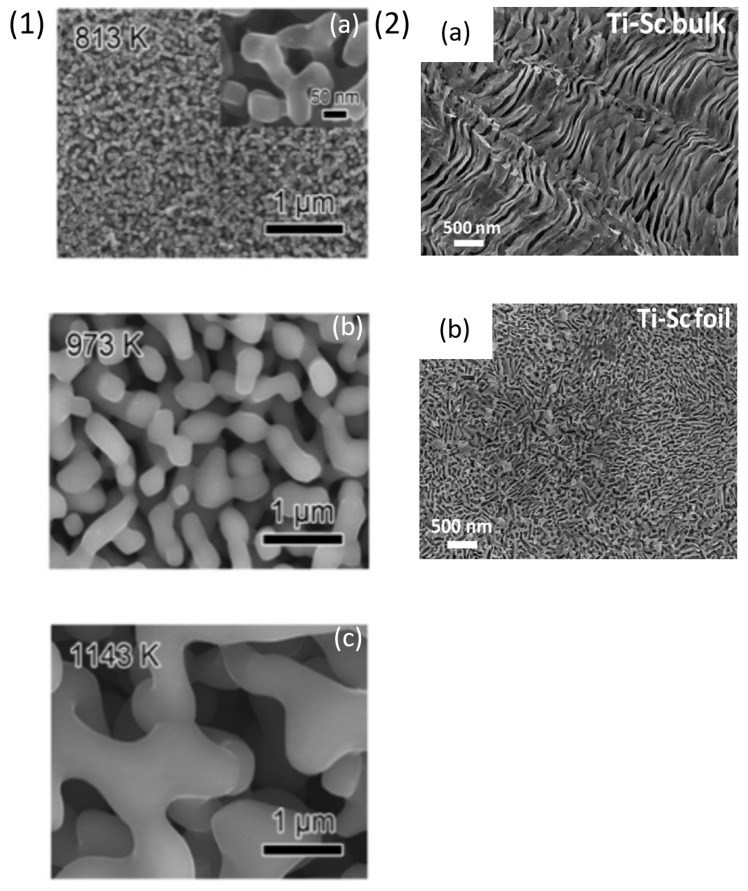
(**1**) [86] SEM images of porous (Ti_0.847_Zr_0.056_Cr_0.098_)_20_Cu_80_ alloy dealloyed in Mg melt for 5 s at (**a**) 813 K (**b**) 973 K (**c**) 1143 K. (**2**) [87] SEM images of (**a**) etched Ti_50_-Sc_50_ bulk alloy (**b**) Ti-Sc etched foil with np-α-Ti.

S. Berger et al. [85] synthesized porous TiMo based alloy using liquid melt dealloying technique with low Young’s modulus and large deformability. In this work the authors fabricated rods of Ti_47.5_Mo_2.5_Cu_50_ at % alloy by arc-melting and suction casting in pure Ar atmosphere. Samples of 1 mm diameter were cut from the rods and then immersed in liquid Mg at two different temperatures of 1073 K and 1173 K for 10 min and 5 min, respectively. During the immersion of the precursor alloy in Mg melt, Cu diffused in Mg melt to form Cu-Mg rich phase whereas Ti and Mo atoms rearranged to form small ligaments and nanocomposites of Ti-rich and Mg-rich phase. Later the Mg-rich phase was etched in 3 M HNO_3_ for 24 h. The dealloyed sample showed presence of α + β Ti phases in XRD. Mo in the alloy acted as a β-isomorphous element for stabilizing the β-Ti phase and being immiscible with Cu, it provided an advantage for producing TiMo phases in the alloy. The SEM images of the untreated samples showed a microstructure with distinct dendrites and ligaments present at the inter-dendritic space due to the immiscibility of Mo with Cu. The average ligament size of the dealloyed samples at 1073 K (TiMo 1073 K) and 1173 K (TiMo 1173 K) was measured to be around 0.75 ± 0.17 µm and 0.53 ± 15 µm and the thickness of the dendrites was 0.94 ± 0.36 µm and 1.92 ± 0.62 µm, respectively. The chemical composition of dendrites was analysed using EDX—TiMo 1073 K and TiMo 1173 K were rich in Ti_94.7_Mo_5.3_Cu_0_ and Ti_95_Mo_4.6_Cu_0.4_ at%, respectively. EDX map of dealloyed samples showed that the ligaments were composed of α-Ti phases, while the dendrites were composed of Mo and β-Ti phases. The concentration of residual Cu was less than 0.5 at % which can be beneficial for application in biomedical implants since low concentration of Cu shows antibacterial effect. T Wada et al. [86] prepared np-multicomponent β-Ti alloy by dealloying in Mg based metallic melt. (Ti_0.847_Zr_0.056_Cr_0.098_)_20_Cu_80_ at% alloy was developed by using arc-melting process followed by melt-spinning to obtain ribbons of 10 mm width and 40 µm thickness. Pure liquid Mg was induction heated in a carbon crucible and ribbon samples were immersed for 5–1800 s with different treatment temperatures, i.e., 813 K, 973 K, 1143 K. After the dealloying treatment the samples were immersed in 3 mol/L of HNO_3_ for 30 min at room temperature. The authors observed that Cu atoms from the alloy selectively dissolved in the melt because the analytical composition of the dealloyed sample detected by EDX was Ti_86.09_Zr_4.73_Cr_9.18_ at %. XRD analysis showed formation of β-Ti and α-Mg composite. Moreover, the chemical etching in HNO_3_ selectively removed α-Mg phases without degrading the porous β-Ti alloy. This study showed that porosity can be controlled by changing the treatment time and diffusion of atoms at solid–liquid interface. As shown in Figure 6 (1) (a–c), different np-morphologies were produced dealloying Cu atoms from the precursor alloy for different treatment times.

Dealloying of Ti alloys by using chemical methods was attempted by N.T. Panagiotopoulos et al. [87] where they produced np-Ti from a Ti_50_Sc_50_ alloy precursor. Due to a wide miscibility gap between Ti and Sc as well as decomposition occurring below 1353 K, a two phases structure was obtained composed of a Ti-rich and Sc-rich phases. Ti_50_Sc_50_ at% bulk alloys were prepared using arc-melting technique and foils were prepared by rapid solidification by melt-spinning obtaining different microstructures, i.e., finer spinodal decomposed Sc-rich part during rapid solidification with respect to arc melting. The samples were dealloyied at room temperature for different times in 70% HNO_3_ and the etching of the less noble Sc-rich phase from the sample was observed, with the formation of a nanoporous Ti-rich phase. The morphologies obtained from the bulk sample and the rapidly quenched sample are reported in Figure 6 (2) (a) and Figure 6 (2) (b), respectively. It is worth noting that the Ti-rich phase was further dealloyied after the complete dissolution of the Sc-rich phase. The EDX and XRD spectra of the dealloyed samples showed presence of np-structures of α-Ti phase with 5 at % Sc after 20 h of treatment time. The study of porosity evolution versus etching time with respect to change in Sc content from 50 at % to 5 at % showed an interdependent trend, i.e., increasing the immersion time resulted in an increment in porosity in vol % and simultaneously a reduction in Sc content. These np-Ti can be used for storing antibacterial drugs, proteins, and bioactive compounds. These materials can also be studied for increasing bone cell adhesion and other beneficial applications for medical implant.

Saba Farhad et al. [88] synthesised Ti and TiMo alloys with 3D-hierarchical porous structures constituted by 400 μm pores and interconnected 10–30 μm micro-pores with nanoporous pore walls with 10–50 nm pores. The processing route includes the use of NaCl spacer and dealloying method, to enable the bimodal distribution of pore size. In detail, powders of pure Ti and Ti_15_Mo_85_ wt % (produced by ball milling) were mixed with Cu powder in 30:70 ratio to produce Ti_30_Cu_70_ and (TiMo)_30_Cu_70_ alloys, respectively, using wet milling in hexane. Afterwards, the powders were mixed with 200–400 µm size NaCl powders in different amounts (40, 50 and 60 vol %) and sintered at 780 °C. Finally, the samples were immersed in hot water (80 °C) for 12 h to dissolve NaCl space holder. The solid-state dealloying process was conducted by using Mg powders mixed with Ti_30_Cu_70_ and (TiMo)_30_Cu_70_ alloys, then cold-pressed at 50 MPa and heat treated at 600 °C for 30 min. During solid-state dealloying, Cu diffused into Mg forming, beside porous Ti or TiMo, CuMg_2_ phase that was finally removed by etching the samples at room temperature in 1 M nitric acid for 10 min. The produced np-Ti and np-TiMo samples were immersed into NaOH solution at 60 °C for 24 h followed by a heat treatment at 600 °C for 1 h. Two groups of np-Ti and np-TiMo samples were considered one with heat treatment and one without heat treatment, respectively, and immersed in SBF (simulated body fluid) for 7 days for measuring apatite formation. Interestingly, the np TiMo samples after heat treatment were able to enhance apatite formation because of higher surface energy of the sample and presence of TiO_2_ layer post heat treatment. Moreover, the resultant samples have acceptable mechanical properties for biomaterial applications. Moreover, the synthetised hierarchical porous structure can enable a fast bone-like apatite nucleation after immersion in simulated body fluid (SBF). The apatite formation is enhanced when TiMo samples are immersed in NaOH and heat treated, another property in favour of an application of these materials for implant applications.

**Figure 7:**
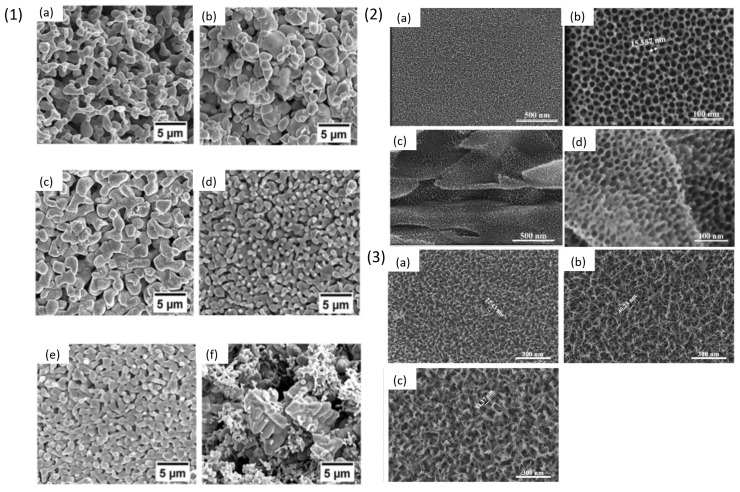
(**1**) [89] SEM images of porous alloys of (**a**) Ti_35_, (**b**) Ti_58_, (**c**) Ti_62_, (**d**) TiZr_49_, (**e**) Zr_73_ and (**f**) TiNb_41_. (**2**) [94] SEM images of nTLM (**a**,**b**) and m/nTLM (**c**,**d**). (**3**) [95] SEM images of electrochemically dealloyed TAV alloy at 2 V in (**a**) 1 M (**b**) 2 M and (**c**) 10 M NaOH.

I V Okulov et al. [89] fabricated metal-polymer composites by combining liquid metal dealloying and infiltering polymer to obtain composites of stiffness matching with human bone. The resulting surface was studied for understanding the changes in mechanical properties before and after polymer impregnation in the porosities. This work establishes a benchmark for future research work for producing np-metal/polymer composites with tunable mechanical properties. This study also comprehensively discussed the role of precursor alloy composition in dealloying. The authors produced crystalline rods of Ti_20_Cu_80_, Ti_30_Cu_70_, Ti_40_Cu_60_, Ti_15_Zr_15_Cu_70_, and Ti_22.3_Nb_7.7_Cu_70_ in at % and a bulk metallic glass Cu_47.5_Zr_47.5_Al_5_. The samples were heat-treated in Mg melt at 1073 K for 300 s which selectively dissolved Al and Cu from the surface of the as-cast rods leaving nanoporous Ti, TiZr or TiNb along the interface of precursor alloy and Mg melt. Afterwards, samples were chemically etched using 3 M HNO_3_ for 5 h to selectively remove residual Mg-rich phases and obtain nanoporous samples (Figure 7 (1) (a–f)). The porous samples were impregnated with polymers, bisphenol F epoxy resin and bisphenol A epoxy resin in vacuum condition for 20 min at 328 K following with 48 h treatment at room temperature to obtain two different polymer impregnated composites. The mass density of the samples was increased after impregnating it with polymer along with the mechanical properties like strength and elastic modulus which involved combining porous metal with medical grade polymer to use as scaffolds. The composite with polymers showed high potential for use as a biomedical device for osseointegration.

In another study by IV Okulov et al. [90] the authors produced nanoporous Ti by using liquid melt dealloying of Ti_50_Cu_50_ at % precursor alloy. The samples were immersed in liquid Mg at 1073 K for 20 min producing Mg_2_Cu phase along the interface of nanoporous α-Ti. The Mg reach phase was etched in 3 M HNO_3_ solution for 5 h to produce np-Ti sample. The author’s investigated the mechanical properties of np-Ti that showed Young’s modulus of 3.3 ± 0.3 GPa and yield strength of 35 ± 3 MPa. The authors correlated these findings using micromechanical modelling which suggested that the localization of porosity present homogeneously in the matrix or along the interface in the composite contributed to overall change in the mechanical behaviour. These findings help in understanding the mechanical contribution of the phases formed during the dealloying process, helping in the production of biomaterials with low modulus.

AV Okulov et al. [91] produced three metal polymer composites composed by Ti_20_Hf_20_Cu_60_, Ti_25_Hf_10_Cu_60_, Ti_30_Hf_10_Cu_60_ alloy (at %) with liquid Bisphenol F epoxy using dealloying in Mg liquid melt at 1023 K for 600 s in carbon crucible. These composites are new class of material with mechanical properties similar to human cortical bone. The composites produced by dealloying followed by polymer impregnation showed intermediate strength of 215–266 MPa and stiffness values between 15.6–20.8 GPa. The impregnation of polymer into dealloyed samples allowed densification of the porous structure and stabilized the Young’s modulus. It is known that molten Mg possesses some limitations for dealloying due to its explosive and flammable nature when used in pure form and hence, it is necessary to use molten Mg in special equipment with an inert atmosphere. Therefore, to overcome this limitation, F. Zhang et al. [92] developed a new method to perform dealloying using Mg powder. In this work, the authors used solid-state dealloying where TiCu alloy was fabricated with spark plasma sintering (SPS) technique. This technique is an electric field assisted process which utilizes DC pulse. Wherein Ti and Cu powders are densified in minutes compared to hot pressing and sintering techniques which requires more times. Ti and Cu powders (30:70 in atomic ratio) were mechanically alloyed using wet ball-milling technique with hexane. The powder mixture of Ti and Cu was then sintered using SPS technique at 373 K and 80 MPa uniaxial pressure for 5–30 min in a vacuum condition. The samples were immersed in the Mg powder in 10:1 ratio (Mg:Ti_30_Cu_70_) and cold pressed at 50 MPa followed by a heat treatment at 773–873 K for 10 min in Ar atmosphere to enable solid-state dealloying process. The XRD analysis of the samples after heat treatment confirmed the formation of α-Ti, Ti_2_Cu and Cu_2_Mg. The presence of Cu_2_Mg phase confirmed the diffusion of Cu with Mg powder as expected in solid-state dealloying treatment. Later, selective corrosion was performed to remove Mg-rich phases from the sample surface using 1 M HNO_3_ for 10 min at room temperature. The α-Ti foam was developed by the phenomenon of solid-state interdiffusion which showed surface area of 34.4 ± 0.8 m^2^/g and pore size of 2–50 nm measured using BET (Brunauer Emmett and Teller). It was observed that the ligament size increased by increasing the heat treatment temperature. On the other hand, properties like hardness and elastic modulus decreased linearly with increasing ligament size, from 259 to 178 MPa and 4.15 to 3.04 GPa, respectively. The authors performed the dealloy process using two different Mg precursors, one as Mg powders and other Mg plate. It was noted that Mg powders were more effective as a dealloying agent, compared to the Mg plates, due to its ease of implementation since the samples do not necessarily need to be fixed tightly with the Mg powder. Additionally, it has a faster reaction time (10–60 min), whereas the reaction time with Mg plates was slower (6–72 h). The authors discussed the significance of nanopores for loading bioactive compounds to prevent implant infection.

Advances in the dealloying technique are reported by Binbin Kang et al. [93] that sputter-coated 2 µm thick film of Ti_25_Nb_15_Cu_60_ (wt %) on Ti_64_Nb_24_Zr_4_Sn_8_ (wt %) alloy. The authors dealloyed Cu from the precursor film using a solution of HNO_3_ and alcohol in 0.08 mol/L at room temperature for 1.5 h and 2 h. The as-dealloyed samples showed reduction in concentration of Cu to 12 wt % after 1.5 h and Cu was fully dealloyed after 2 h. The dealloyed film showed elastic modulus near 24 GPa and hydrophilic interaction.

Lan Wang et al. [94] studied in vitro biocompatibility of dealloyed Ti_3_Zr_2_Sn_3_Mo_25_Nb alloy. This type of alloy has been studied for potential orthopaedic implant application, due to its low elastic modulus (50–80 GPa), high specific strength and good plasticity. In this study the authors compared electrochemical dealloying results of two types of alloys, i.e., cold-rolled with elongated grains composed of primary metastable α, β and α″ phases (m/n-TLM) and solution-treated at β-transus temperature to produce mostly β phases and small concentration of α″ phase (n-TLM). Samples were dealloyed in a mixed acid electrolyte (0.2% HF, 2% HNO_3_) at 3 V for 1 h as shown in Figure 7 (2) (a–c). The dealloyed samples showed hydrophilic interaction with water and mean pore diameter around 15.6 nm ± 2.3 nm for n-TLM while m/n-TLM samples showed wide groves of nearly 1.5 µm length and 0.4 µm width. The dealloying thickness of n-TLM surface was found to be roughly 70 nm measured using XPS technique via sputter depth profile. Surface composition using XPS showed the sample surface to be rich in TiO_2_ layer. The dealloyed samples were investigated for biocompatibility using mouse osteoblast cell line (MC3T3-E1). Another study, by Lan Wang and his group [95] reported the use of electrochemical dealloying for reducing Al concentration on widely used biomedical implant material Ti6Al4V using NaOH solution with 1, 2 and 10 M solution. Increasing the concentration of the NaOH solution the potentiodynamic polarization curves of pure Al, pure Ti and Ti6Al4V alloy were shifted negatively and the current density drastically increased. 1 M solution was used to study the effect of dealloying with potential ranging from 0.8 V, 1.4 V, 2 V to 3 V for 1 h. The rate of dealloying increased by increasing potential and above 2 V the surface showed formation of 3D grid structures. The samples dealloyed at 2 V for 1 h with different concentrations like 1 M, 2 M and 10 M showed evolution of porosity wherein pore size increased with increasing the electrolyte concentration as shown in Figure 7 (3) (a–c). The contact angle of the dealloyed samples reduced with increasing the concentration of electrolyte making the surface more hydrophilic. The three selected electrochemically dealloyed samples were further studied to understand their biocompatibility using mouse osteoblast cell line (MC3T3-E1), further discussed in Section 4.

### 3.2. Ti-Based Bulk Metallic Glasses

Dealloying techniques have been extensively used for crystalline alloys to develop np- structures. However, metallic glasses provide better opportunity as a precursor alloy for dealloying when compared with crystalline alloys. This class of material possesses many advantages due to the presence of a monolithic amorphous phase with a homogeneous composition [96]. In a study by F. Scaglione et al. [97] crystalline and amorphous multicomponent Au-based alloys were investigated for understanding the dealloying mechanism by free immersion in HNO_3_ solution. The authors observed that in crystalline alloy during dealloying, the more noble adatoms move by surface diffusion following the original grain pattern whereas in amorphous alloy, due to absence of crystalline grains, Au adatoms aggregate in randomly oriented nanocrystals. In both cases, the ligaments produced were crystalline.

**Figure 8:**
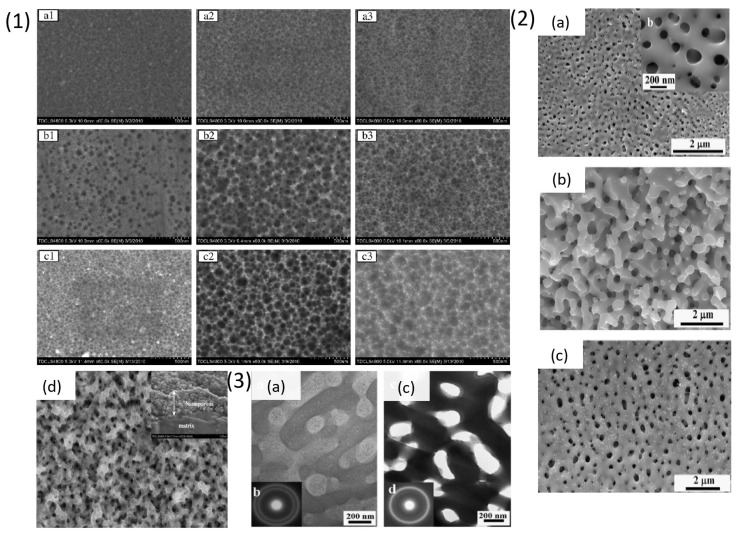
(**1**) [98] SEM images of dealloyed Ti_30_Cu_70_ ribbon samples with varying concentration of HNO_3_ (**a1**) 3.38 mol/L, (**a2**) 5.36 mol/L and (**a3**) 7.55 mol/L; temperature (**b1**) 333 K, (**b2**) 343 K (**b3**) 353 K and; potential (**c1**) 0.6 V (**c2**) 1 V (**c3**) 1.4 V. (**d**) SEM image of Ti_30_Cu_70_ porous sample treated with 5.36 mol/L HNO_3_ at 1 V and 343 K for 180 min. (**2**) [99] SEM images of porous Y_20_Ti_36_Al_24_Co_20_ ribbon samples at (**a**) low magnification (**b**) high magnification (**c**) cross-section of the sample chemically dealloyed in 0.1 M HNO_3_ for 24 h (**d**) after electrochemical dealloying in 0.1 M HNO_3_ at 1.9 V for 30 min. (**3**) [99] TEM images of Y_20_Ti_36_Al_24_Co_20_ alloy (**a**) before dealloying with electron diffraction pattern (**b**) (inset) and (**c**) after dealloying with electron diffraction pattern (**d**) (inset).

This section discusses all the dealloying attempts made on Ti-Cu based metallic glassy alloy to fabricate np-structures of titanium. S. Zhu et al. [98] dealloyed three different compositions of amorphous alloy, i.e., Ti_30_Cu_70_, Ti_40_Cu_60_ and Ti_50_Cu_50_ at %, using HNO_3_ solution. The authors studied influence of dealloying parameters like applied potential, electrolyte concentration and temperature. The alloys were produced by arc-melting and then melt-spun into amorphous ribbons that were electrochemically dealloyed using Pt mesh as a counter electrode and saturated calomel electrode as the reference. The samples were treated with different concentrations of HNO_3_, reaction time and temperature, to remove Cu and to form a dense TiO_2_ layer. It was observed that lower concentration of HNO_3_ possesses lower corrosive and oxide formation ability. In Figure 8 (1) (a1) results obtained from dealloying Ti_50_Cu_50_ at % in different concentrations of HNO_3_ solution at 1 V and 343 K for 80 min have been provided. The sample showed formation of smaller pores in 3.38 mol/L HNO_3_, which later stopped due to formation of a dense TiO_2_ oxide layer. In Figure 8 (1) (a2,a3), it was observed that increasing the concentration of the HNO_3_ solution to 5.36 mol/L and 7.55 mol/L led to formation of homogeneous pores of about 15–20 nm size. As shown in Figure 8 (1) (b1–b3), the Ti_40_Cu_60_ alloy was dealloyed using 5.36 mol/L HNO_3_ at 1 V for 90 min at different temperatures of 333 K, 343 K and 353 K. The results from this treatment suggested that there is an increment in the number of pores with increasing temperature because the systems energy is enhanced at high temperature which leads to acceleration in diffusion of atoms. In Figure 8 (1) (c1–c3) morphology of Ti_40_Cu_60_ alloy treated in 5.36 mol/L HNO_3_ solution at 353 K for 180 min at different potentials of 0.6 V, 1 V, 1.4 V is shown. The depth and diameter of the pores increases with increasing the applied load voltage, which shows that load voltage is one of the most important parameters for dealloying. However, at 1.4 V (Figure 8 (1) (c3)) there were pit formations instead of dealloying due to presence of Ti ions. This range of potential promotes excessive passivation making it difficult for the electrolyte to eliminate Cu from the surface. The ideal np-structure was obtained on Ti_30_Cu_70_ ribbon with 50 nm diameter and 100 nm pore wall thickness, when treated with 5.36 mol/L HNO_3_ at 1 V and 343 K for 180 min as shown in Figure 8 (1) (d). The np-surface had depth of 500 nm and was composed of TiO, Ti_2_O_3_, TiO_2_ and Cu_2_O oxides detected using XPS technique. The authors observed a dense passive film formation of titanium oxides credited to the treatment in HNO_3_ solution which prevents the corrosion of Ti while Cu reacts with the HNO_3_ solution.

Phase-separated Ti-based metallic glass ribbons containing Ti-rich and Y-rich phases were chemically and electrochemically dealloyed by J. Jayaraj et al. [99]. The authors developed metallic glass ribbons by melt-spinning. Three different alloy compositions, i.e., Y_56_Al_24_Co_20_, Ti_56_Al_24_Co_20_, and Y_20_Ti_36_Al_24_Co_20_ were developed containing Ti-rich (Ti_56_Al_24_Co_20_) and Y-rich (Y_56_Al_24_Co_20_) amorphous monolithic phases, while the quaternary alloy, Y_20_Ti_36_Al_24_Co_20_, contains two interconnected amorphous phases, Ti-rich (Ti_43.3_Y_3.7_Al_15.3_Co_37.7_) and Y-rich (Y_38.8_Ti_12.8_Al_37.1_Co_11.3_). The ribbon samples were dealloyed in 0.1 M HNO_3_ solution via chemical and electrochemical routes. The authors measured the corrosion current (Icorr) and corrosion potential (Ecorr) by using the Tafel slope to understand the dissolution potential of the sample where the Y-rich phase acts as the anodic part and is leached without affecting the Ti-rich phase which is the cathodic part. The SEM images in Figure 8 (2) (a,b) show the 3-D interconnected porous surface morphology of the sample dealloyed for 24 h in 0.1 M HNO_3_ using chemical treatment and Figure 8 (2) (e) shows its cross-section indicating that the dealloying was achieved homogeneously throughout the depth of sample. Figure 8 (2) (d) shows the sample electrochemically dealloyed at 1.9 V for 30 min, a porous morphology was obtained similar to that obtained after chemical dealloying. The two distinct Y and Ti-rich phases were observed in Figure 8 (3) for the Y_20_Ti_36_Al_24_Co_20_ alloy. TEM images of Y_20_Ti_36_Al_24_Co_20_ alloy (a) before dealloying with electron diffraction pattern (b) (inset) revealed two diffused halo rings corresponding to Y-rich phase and Ti-rich phase. After dealloying (Figure 8 (3) (c) with electron diffraction pattern (d) (inset)) shows TEM image of sample after dealloying where only the amorphous ring related to the presence of Ti-rich phase is observed. EDS analysis of chemically dealloyed and electrochemically dealloyed samples revealed the composition of Y_5.4_Ti_56.2_Al_18.2_Co_20.2_ and Y_4.2_Ti_56.5_Al_11.2_Co_28.1_, respectively, which indicated the reduction in the amount of Y and Al while enrichment of Ti and Co concentration.

**Figure 9:**
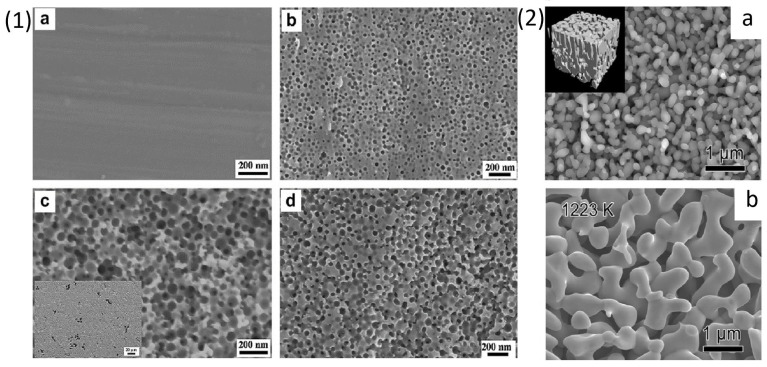
(**1**) [100] SEM images of porous Ti_45_Y_11_Al_24_Co_20_ ribbons dealloyed in 1 M H_2_SO_4_ (**a**) untreated (**b**) dealloyed for 20 min (**c**) dealloyed for 12 h (**d**) dealloyed for 60 min. (**2**) [101] High-magnification SEM images of dealloyed Ti_30_Cu_70_ sample in Mg melt for 5 s (**a**) at 973 K (inset: tomogram image) (**b**) at 1223 K.

J. Jayaraj et al. [100] fabricated np-structures on Ti_45_Y_11_Al_24_Co_20_ ribbons by selectively etching Y-rich phase in 1 M H_2_SO_4_ as shown in Figure 9 (1). Amorphous ribbons were fabricated by using a melt-spinning method and immersed in 1 M H_2_SO_4_ solution for different times (20 min, 60 min and 12 h). The SEM images in Figure 9 show irregular pores after 20 min of treatment whereas increasing the immersion time of 60 min led to an enhancement in the pore size of 40 nm and density with stable oxide layer. However, prolonging the immersion time to 12 h the pore size incremented further making the porous layer highly fragile and eventually collapse. The sample dealloyed for 1 h was heat treated at 673 K for 10 min in Argon atmosphere. The sample surface showed change in colour which was attributed to the formation of a thick oxide layer. The sample also showed enhanced corrosion resistance of 0.0037 MPY (miles per year) in simulated body fluid (Ringer’s solution) due to the presence of the oxide layer, which is 18 times lower as compared to the conventional Ti6Al4V alloy.

T. Wada et al. [101] reported a dealloying method for preparation of less-noble metals using Mg metallic melt to produce structures which are self-organising for various applications like fluid filters, gas absorption media, biomaterials, etc. Ribbons of Ti_30_Cu_70_ alloy were prepared using melt-spinning and then immersed in liquid Mg inductively heated at two different temperatures (973 K, 1223 K) for 5 s. Then, the samples were etched in 3 mol/L HNO_3_ for 30 min at room temperature to remove Mg reach phase. The SEM images in Figure 9 (2) show 3-D connected Ti granules of 200 nm size and the tomogram confirms presence of bi-continuous porous structure with 47% porosity and surface area of 3.9 × 10^3^ m^2^g^−1^. The samples treated at 1223 K were coarser in morphology with respect to the samples treated at 973 K because at low temperature the diffusion of atoms at alloy/Mg melt is slower which leads to the formation of finer np-structures as shown in Figure 9 (2) (a,b).

#### Pseudo-Dealloying (PDS) in Metallic Glasses

Recent studies have shown that the pseudo-dealloying (PSD) treatment on the surface of metallic materials can enhance their properties for biomedical applications. Specifically, this process has been investigated on amorphous titanium-based alloys and involves the application of selective chemical or electrochemical dealloying methods, which allow for the dissolution of copper, an excess element in this type of alloy, while simultaneously forming a nanoporous Ti/Zr oxide layer.

Tiwari et al. demonstrated the effectiveness of an ammonia-based solution in pseudo-dealloying to enhance the biocompatibility of a Ti-Cu-Zr-Fe-Sn-Ag amorphous alloy [102]. This treatment resulted in hydrophilic and hemocompatible surfaces, making the material suitable for blood-contacting medical devices. Furthermore, the chemically treated Ti-based amorphous alloy exhibited cytocompatibility with Saos-2 and HOb cells, as well as antibacterial activity against Pseudomonas aeruginosa. The sample chemically dealloyed for 60 min in a solution of NH_4_OH, H_2_O_2_, and H_2_O in a 5:1:4 vol ratio demonstrated significant potential for medical implant applications, showing a favourable balance between biofilm resistance and cytocompatibility [103]. In a subsequent study [104], Tiwari achieved improved biocompatibility for implant applications through surface modification with nitric acid, which also facilitates the generation of reactive oxygen species, thereby preventing bacterial contamination.

Electrochemical pseudo-dealloying has been investigated in three key studies. Shtefan et al. extensively examined the pitting corrosion of two Ti-Cu based glassy alloys in chloride-containing solutions, revealing distinct corrosion behaviours due to compositional differences. Their work highlighted the critical role of alloying elements like Pd and Cu in determining corrosion resistance [105]. In a separate study [106], the electrochemical pseudo-dealloying of Ti_40_Zr_10_Cu_36_Pd_14_ bulk metallic glass (BMG) was shown to reduce copper content, enrich palladium, and form a nanoporous layer that enhances both corrosion resistance and biocompatibility. Additionally, Fernández-Navas et al. advanced Ti-based bulk metallic glasses through electrochemical pseudo-dealloying, further improving corrosion resistance and reducing surface copper content, thus increasing their suitability for implant applications [107].

### 3.3. Ti-Based Shape Memory Alloy

**Figure 10:**
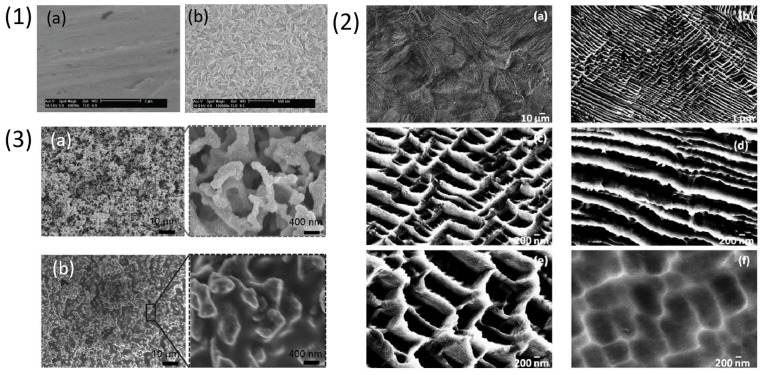
(**1**) [111] SEM images of NiTi alloy (**a**) before and (**b**) after dealloying for 15 h in nitro dioctyl phthalate, H_2_O, HCl and H_2_SO_4_ solution. (**2**) [112] SEM image of chemically dealloyed NiTi alloy (**a**) at low magnification (**b**) at high magnification up to one grain (**c**) showing 2-D networked structure (**d**) at high magnification showing 1-D channel (**e**) after annealing at 673 K for 1 h (**f**) after annealing and passivation. (**3**) [113] SEM image of NiTi alloy sample (**a**) after electrochemical dealloying at 1.82 V in 2 M HNO_3_ (**b**)showing porosity after polymer coating, PNHNC-2.

Titanium-based shape memory alloys like TiNi are used as biomedical materials due to their impressive properties like super elasticity, shape memory effect related to their high strength, low elastic modulus and high strain recovery because of reversible martensitic transformation. TiNi alloy exhibits better biocompatibility compared to stainless steel and enhanced corrosion resistance than Co-Cr alloy when studied in an artificial saliva [108]. However, high concentration of Ni is a cause of concern. The corrosion of Ni-based alloys in the body environment can produce Ni ions which when dissolved in large amounts cause negative responses like sensitization, carcinogenicity, teratogenicity, headaches and dyspnoea to the patients [109,110]. To overcome this problem several attempts have been made to modify the surface of NiTi alloy. One such attempt is using the dealloying technique where J. Huang et al. [111] investigated biocompatibility of NiTi shape memory alloy by dealloying at low temperature to obtain np-TiO_2_ layer and remove Ni from the surface using chemical dealloying in nitro dioctyl phthalate, H_2_O, HCl, H_2_SO_4_ in 4:1:2:3 volume ratio for 4, 6, 9 and 15 h. The samples were stirred at 323 K for 15 h and annealed at 673 K for one h followed by a passivation process in HNO_3_ (30%). The XRD pattern showed diffraction peaks of Ti phase, and rutile phase which appeared after the treatment of the samples showing the formation of TiO_2_ layer. The SEM images in Figure 10 (1) (a,b) show surface of non-dealloyed and dealloyed samples, respectively. The authors observed changes on the surface with varying time of etching. Initially, the surface of the samples began to etch after 4 h of treatment and at 15 h the sample surface showed presence of nano-grid structures due to dissolution of Ni phase. The dealloyed ribbon samples showed enhanced corrosion potential in Hank’s solution with pH 7.45. The results of cell studies will be discussed in Section 4.

Another article on NiTi by Manjunath Chembath et al., [112] discussed the results of chemically treated NiTi alloy using FeCl_3_ solution. Three different types of treatment processes were used to prepare the final sample which involved chemical treatment in FeCl_3_, heat treatment and passivation. The samples were immersed in 0.18 M FeCl_3_ solution for 1 h at 343 K followed by annealing at 673 K for one h and passivation in 30% HNO_3_. From the ICP analysis of the dealloyed samples, it was observed that in the presence of FeCl_3_ the content of Ni present in the alloy was lowered and Ti remained inert. The wetting behaviour of the surface was super-hydrophilic while untreated sample showed 85.6° contact angle. The samples were tested for formation of hydroxyapatite by immersion in Hank’s solution for 14 days and the formation of globular nodules on the sample surface was observed, confirmed to be hydroxyapatite particles in EDX analysis, whereas untreated NiTi alloy showed no growth of hydroxyapatite after 14 days of immersion. The morphology of chemically treated surfaces showed roughness due to the presence of nano-grid structures as shown in Figure 10 (2) (a). The grid structures were found to vary from one grain to another. For instance, in Figure 10 (2) (b) presence of long 1-D channels localized at the grain boundaries is observed and in Figure 10 (2) (c), 2-D network exists with grid wall length of 50–100 nm whereas the inner grid spaces are of 1 µm. It was concluded that surface area of the sample was increased by 17% after treatment in FeCl_3_. The samples showed enhanced properties like good corrosion resistance in Phosphate-Buffered Saline (PBS) and bioactivity with better osseointegration.

Dongmian Zang et al. [113] developed NiTi/Hydrogel nanocomposite with an antibiofouling surface to avoid undesirable adhesion of platelets to prevent blood thrombosis and coagulation. In this research electrochemical dealloying technique was used to fabricate a bicontinuous porous layer on NiTi alloy (PN) of 150 µm thickness at 1.82 V in 2 M HNO_3_ for 3 h. This led to successful dissolution of Ni and oxidation of Ti as shown in SEM images (Figure 10 (3) (a)). The treatment was followed by in situ photopolymerization by immersion in a solution of vinyl phosphonic acid (VPA), polyvinyl alcohol (PVA), N,N′-methylene bis-acrylamide (MBAA), Acrylamide (Aam), glutaraldehyde and VPA to obtain super-oleophobic NiTi/hydrogel nanocomposites (PNHNC). The photopolymerization treatment was performed to obtain a bicontinuous NiTi layer firmly interlocked with the hydrogel forming a composite (Figure 10 (3) (b)). The composite with hydrogel coating possessed 95.1% PNHNC-1, 96.7% PNHNC-2, 97.6% PNHNC-3 content of water which showed decrease in hardness and elastic modulus of the composite when compared with pristine NiTi alloy. The PNHNC-3 with 96.7% of water content remained super elastic in behaviour and possessed strong interfacial adhesion after the treatment processes. The treated samples were studied for platelet adhesion to understand hemocompatibility of the samples, which will be discussed in Section 4.

## 4. Biocompatibility Studies on Dealloyed Samples

**Figure 11:**
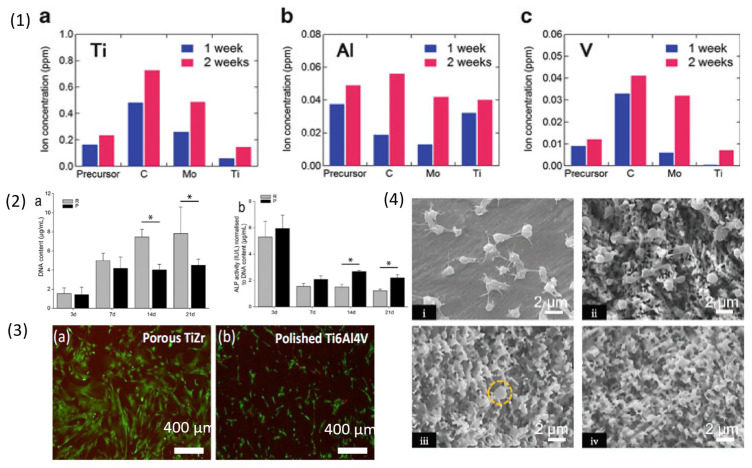
(**1**) [78] ion release study of untreated precursor and dealloyed samples in Ti, C, Mo crucibles for studying release of (**a**) Ti (**b**) Al (**c**) V ions in SBF for 1 and 2 weeks. (**2**) [79] (**a**) results of DNA content and (**b**) ALP activity. (**3**) [82] Fluorescent images of porous (**a**) TiZr and (**b**) T6Al4V alloy as control in HUCPV cell culture. The green staining depicts live cells, and red staining depicts dead cells after 5 days of cell culture. (**4**) [113] shows SEM images of adhered platelets on (**i**) pristine NiTi alloy, (**ii**) PN, (**iii**) PNHNC-1, (**iv**) PNHNC-2, (**v**) PNHNC-3, respectively.

This section discusses the results of biocompatibility tests of the reported articles in Section 3. Yuichi Fukuzumi et al. [78] studied dealloying treated on Ti6Al4V alloy in three different crucible materials made up of C, Mo, Ti and investigated ion release (Ti, Al, V) in simulated body fluid (SBF) for 1 and 2 weeks from samples after dealloying (Figure 11 (1)). Release of Al ion from the dealloyed samples was low in all cases, while the samples dealloyed in C and Mo crucibles showed higher Ti and V ion release, maybe due to presence of porous structure with high surface area. The samples dealloyed in Ti crucible showed reduced ion release which could be due to less porosity on the sample surface. It was concluded that crucible material influences the composition and morphology of the alloy.

I V Okulov et al. [79] dealloyed T6Al7Nb alloy with Mg melt to produce Ti rich phase on the sample surface. Biocompatibility of the dealloyed surface was investigated using human umbilical cord perivascular cells. To understand the influence of material used in the experiment, the amount of DNA (Deoxyribonucleic acid) contents was measured from the cell culture after 3, 7, 14, 21 days as shown in Figure 11 (2) (a). ALP activity (Alkaline activity), a biochemical marker responsible for bone formation and mineralization, was high on the porous samples at day 14 and 21 of the culture (Figure 11 (2) (b)). Dealloyed samples were more suitable for cell differentiation compared to the non dealloyed sample. Cytotoxicity test on 3, 7, 14, 21 days cultured samples stained using calcein (green-alive cells) and ethidium homodimer-1 (red-dead), showed that porous dealloyed samples had good coverage with live cells, i.e., exhibit good cytocompatibility.

I V Okulov et al. [82] performed cytocompatibility test on Ti-Zr alloy using human umbilical cord perivascular cells (HUCPV). LIVE/DEAD assay at day 5 days was performed and commercial Ti6Al4V alloy was used as a control sample (Figure 11 (3)). It was observed that porous materials with roughness were more favourable to cell adhesion compared to polished sample surface. The samples were biocompatible with less red stained dead cells on porous Ti-Zr compared to the polished surface of Ti6Al4V alloy, making it a potential candidate as an implant material.

Jin Huang et al. [105] studied cellular compatibility on TiO_2_ coated NiTi shape memory alloy by using Dermal Mesenchymal Stem Cells (DMSC). The study was performed at day 1, 5, 8 on two groups, Group A: treated samples, Group B: untreated samples. Results showed enhanced cell proliferation on Group A samples due to the presence of TiO_2_ layer which can prevent release of Ni ions in the system. The two groups were studied for presence of hydroxyproline which helps in understanding the collagen synthesis and alkaline phosphatase for understanding cell growth, the results of hydroxyproline study showed slow cell activity in group B because the content was gradually reducing from 12.2 mg/L at day 1 to 6.5 mg/L at day 8, while group A samples showed low adsorption of hydroxyproline compared to group B with 5.5 mg/L at day 1 to 2.8 mg/L at day 8 which explains high cell growth on group A. The alkaline phosphatase analysis in cell culture medium showed decline in the content for groups A, i.e., the samples have less influence on the cell metabolism due to presence of passivated surface promoting slower release of Ni ions. In the cell culture medium release of Ni ion was studied for day 5 and 8 to understand the efficiency of TiO_2_ layer on NiTi alloy. The group A after day 8 of study showed 3.7 times lower Ni ion release when compared to group B. This study paves a new path for NiTi alloy which can be used for implant application.

Dongmian Zang et al. [107] investigated platelet adhesion on dealloyed NiTi samples by platelet suspension method. The SEM images in Figure 11 (4) showed that the platelets were activated, and their pseudopods were spread around on the NiTi sample surface. The composites were also studied for protein adsorption using BSA-FITC (fluorescein isothiocyanate labelled bovine serum albumin), the result showed that the amount of adsorbed BSA-FITC was inhibited on treated samples when compared to pristine alloy. The wettability test on the treated samples showed low surface adhesion force with isooctane oil proving it to be super-oleophobic (contact angle above 150°). The composite samples showed lower modulus compared to the untreated NiTi alloy suggesting potential application as endovascular stents.

**Figure 12:**
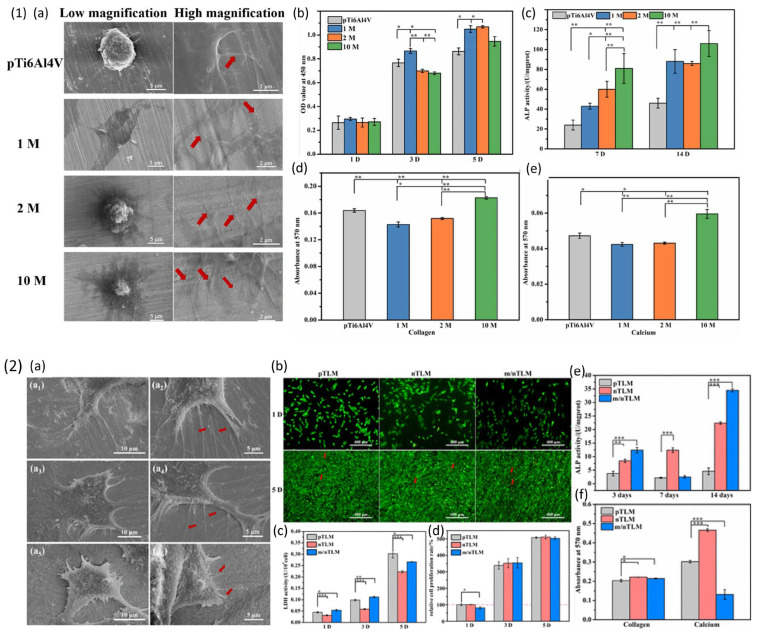
Cytocompatibility evaluation of Ti6Al4V and TLM dealloyed samples: (**1**) (**a**) SEM images of cell morphology after being cultured for 6 h; (**b**) cell proliferation on different surfaces estimated by cck-8 assay for 1, 3, 5 day; (**c**) the degree of ALP activity; (**d**) the quantification of collagen secretion; (**e**) the quantification of calcium deposition, (* represent *p* < 0.05, ** represent *p* < 0.01). (**2**) [94] (**a**) two examples of SEM micrograph of the MC3T3-E1 cells cultured on pTLM (**a1**,**2**), nTLM (**a3**,**4**), and m/nTLM (**a5**,**6**) samples after 6 h of incubation (arrows indicating filopodia), (**b**) after 1 and 5 days, cultured of MC3T3-E1 stained with two well-described probes, indicating live cells (green) and dead ones (red), arrows indicate the dead cells; (**c**) LDH activity for 1, 3 and 5 days; and (**d**) cell proliferation measured by the AlamarBlue assay for 1, 3, and 5 days, * represents *p* < 0.1, ** represents *p* < 0.05, and *** represents *p* < 0.05. (**e**) ALP activity at day 3, 7, and 14 of incubation, (**f**) collagen and calcium deposition at 28 days of incubation.

A comprehensive invitro study has been performed by Lan Wang and his group on two different class of dealloyed samples discussed in Section 3.1 [94,95]. The authors studied biocompatibility of Ti6Al4V alloy which is widely used for orthopaedic implant application and Ti_3_Zr_2_Sn_3_Mo_25_Nb dealloyed alloy which is considered as a strong alloy candidate for biomedical implant application. Mouse osteoblast cell line (MC3T3-E1) was used for studying cytocompatibility which includes cell cytotoxicity of samples cultured at day 1 and day 5 using lactate dehydrogenase kit (live/dead), cell adhesion and spreading observed using SEM and fluorescence microscopy after 6, 12 h, 1, 3, 5 days and cell proliferation of co-cultured sample after 1, 3, 5 days using alamarblue. Osteogenic differentiation testing which includes alkaline phosphate (ALP) at day 3/7 and 14, collagen secretion (COL) of sample after 28 days and extra cellular matrix (ECM) mineralization for determining calcium deposition (CAL) after 28 days. ALP activity is an indicator of early osteogenic differentiation and CAL and COL activity are indicators of late osteogenic differentiation of osteoblasts leading to fast maturation of bone tissue and it is important for successful bone implant integration. Dealloyed Ti6Al4V samples showed positive response towards cells adhesion at early stage of cell incubation of 6 h with cell spreading area significantly higher on dealloyed samples shown in Figure 12 (1) (a). After day 1, 3, and 5 of incubation the cells showed good anchorage on the surface and high cell proliferation on the dealloyed samples indicating the surface of the samples promoted cell adhering and multiplication. Additionally, the samples were non cytotoxic after day 1 of incubation. The cells showed well attached and stretched filopodia with flat cells because of proper adhesion of cells on the dealloyed surface, compared to control sample with polished surface. The dealloyed sample in 1M NaOH solution showed highest cell proliferation (Figure 12 (1) (b)), while 10 M concentration sample showed high ALP, COL and CAL activity indicating dependent behaviour of cells towards alkaline treated surface (Figure 12 (1) (c–e)). The dealloyed Ti_3_Zr_2_Sn_3_Mo_25_Nb alloys showed high protein adhesion due to roughness on the surface and hydrophilic interaction which is favourable for cells adhesion. At early stage of incubation time of 6 h the cells showed extended and flattened morphology which is well spread using the filopodia in multiple directions on pTLM and nTLM, while on the supple m/nTLM the cells are shrunk with small filopodia extensions as shown in Figure 12 (2) (a). The results of Live/dead test showed a few dead cells on the samples after 5 days of incubation as shown in Figure 12 (2) (b). The dealloyed samples showed lower LDH activity compared to polished sample (Figure 12 (2) (c)) indicating the dealloyed surfaces are less cytotoxic compared to untreated surface. The cells at day 1 showed elongated morphology indicating good cell proliferation due to presence of hierarchical morphology however no significant difference is observed between the dealloyed and untreated samples. On the other hand, the hierarchical surface improved cell proliferation (Figure 12 (2) (d)) and differentiation with ALP activity, collagen and calcium deposition (Figure 12 (2) (e,f)) and still retained the contact guidance function, which implied good bonding for osseointegration.

## 5. Challenges and Opportunities

In Table 2 the alloys and dealloying techniques reported in this review paper with the key findings are reported. 

Dealloying technique is a controlled corrosion method for developing self-organized np- morphology with large surface areas. This method, being one of the very old techniques, still finds its application in emerging fields like energy storage, sensing, catalysts, and biomedical implants made up of Ti alloys. In the past decades researchers have been able to successfully discover new ways to dealloy Ti-based alloys for production of np-Ti and npTiO_2_ layers. Despite these attempts, it is required that more research is done for finding the full potential of this technique. In the current scenario dealloying of Ti faces difficulties of passivation when immersed in chemical solutions due to high stability of TiO_2_ layer, hence, etching of other elements becomes difficult. In case of liquid metal dealloying current work is focused on using Mg melt but it possesses some challenges because handling of Mg is difficult, and it cannot be scaled easily for commercial purposes. Also, the solid-state and vapour-phase dealloying techniques pose certain limitations with the type of dealloying components. These techniques require further optimization and better understanding of the dealloying process in order to tailor the np-structures as per the desired application. The dealloying method can provide new pathways for synthesis of np-Ti-based alloys with multiple components. A. Chuang and J. Erlbacher [114] discussed progress and challenges in integrating dealloying method with additive manufacturing which expands opportunity for scientific community to incorporate these two techniques to make nanocomposites and np-materials with wide selection of elements from the periodic table. The authors further discussed the ongoing research on dealloying of additively manufactured materials produced by direct ink writing like Au-Ag, Ni-Cu using electrochemical route. Several alloys produced with selective laser melting/sintering were treated using dealloying methods. For instance, electrochemical dealloying was used for Cu-Mn, Al-Si, while liquid-melt dealloying was used on Co-Cr-Mn, Fe-Cr-Ni and Nb-Ti, and solid-state and vapour-phase dealloying were used on Fe-Ni and brass, respectively.

Recent investigations into pseudo-dealloying of titanium-based amorphous and bulk metallic Ti-based alloys demonstrate a consistent enhancement of biocompatibility through copper reduction and the formation of nanoporous Ti/Zr oxide surfaces. Both chemical and electrochemical approaches share the core outcome of lowering cytotoxic Cu levels and generating protective Ti/Zr oxide layers, but they emphasize different strengths: chemical pseudo-dealloying, often using ammonia- or nitric acid-based treatments, directly improves hemocompatibility, cytocompatibility with osteoblast-like cells, and imparts antibacterial activity, making it particularly relevant for blood-contacting implants. In contrast, electrochemical pseudo-dealloying emphasizes corrosion resistance and surface stability by enriching biocompatible elements like Pd, which indirectly enhances biological suitability by limiting toxic ion release and ensuring long-term implant durability. Across both methods, a regular trend emerges where the interplay of copper removal, nanoporous structure formation, and surface chemistry modification results in favorable cell interactions, infection resistance, and material longevity, collectively highlighting pseudo-dealloying as a versatile strategy for advancing next-generation biomedical implants.

## 6. Conclusions

Dealloying technique has been extensively studied for production of np-surfaces with unique morphology for various applications like photocatalyst, energy storage, sensing, biomedical implants, etc. The procedures for fabrication of np-Ti alloys and understanding its application for biomedical implants have been discussed in this review paper. Dealloying of Ti-based alloys have gained substantial attention to produce np-Ti and np-TiO_2_ surface by selectively removing one or more components which are undesirable on the sample surface to ensure biocompatibility. This technique is a multidisciplinary research work with immense potential for tailoring the sample surface for use as implant materials. In this review several articles are discussed which used various types of dealloying techniques like chemical, electrochemical, solid-state and liquid melt dealloying. These techniques have a potential to produce Ti or TiO_2_ surface layers on crystalline, amorphous, shape memory, and composite materials. However, the amount of research done in this field is still limited and it is expected to develop more in the future. The aim of this review paper is to attract more attention towards the advantages of dealloying method for biomedical implant application and push more limits in finding better ways to produce np- Ti-based alloys with enhanced biocompatibility. This method can be a promising tool for future aspects as it is inexpensive and scalable. Further optimizations are required in order to scale this method for commercial applications to produce effective Ti-based porous implants.

## Figures and Tables

**Table 1:** The different types of dealloying are summarised with their relevant characteristics. An AB solid solution is defined as the precursor alloy, in which the B element must be removed in order to obtain a nanoporous A metal.

Dealloying Strategies	Dealloying Media	Physicochemical Approaches Employed to Achieve Dealloying	Mechanism of Atoms Removal	Temperature (T)	Thickness of Nanoporous Layer Produced
Chemical/electrochemical dealloying	Electrolyte	Difference in electrochemical potential between A and B. B is the less noble element.	The less noble element B is dissolved into the electrolyte. A moves on the surface by surface diffusion creating ligaments and pores, i.e., forming a nanoporous material	Below electrolyte boiling T. Increasing the T allow an increasing in the dealloying process	It is related to the time of dealloying. By increasing the process time, the dealloyed volume is increased. The whole volume of the material can be dealloyed
Liquid metal dealloying (LMD)	Liquid metal. In most of the cases Mg is used as dealloying medium	Difference in enthalpy of mixing of elements present in precursor alloy with respect to the liquid melt (i.e., the dealloying medium).	B has negative enthalpy of mixing with the metallic melt, so it diffuses into the melt while A (with positive enthalpy of mixing with the melt) remains on the surface of the solid alloy forming a nanoporous metal	Above the dealloying media melting T and below the melting T of the precursor	It is related to the time of dealloying. By increasing the process time, the dealloyed volume is increased. The whole volume of the material can be dealloyed
Solid-state dealloying (SSD) or solid metal dealloying (SMD)	Solid metal	Difference in enthalpy of mixing of elements present in precursor alloy with respect to the dealloying medium.	Solid-state diffusion of B atoms from the AB precursor alloy into the dealloying medium leaving apart nanoporous A. At the end of the process a phase is formed with B and the dealloying metal.	T must be high to increase the process rate, but it must be below the melting T of dealloying medium and precursor	As the process is based on the solid-state diffusion of B atoms, the dealloying rates are low. Therefore, only thin layers can be produced on the surface.
Vapour Phase Dealloying (VPD)	Vacuum	Difference in saturated vapour pressure. B has a high vapor pressure with respect to A.	B evaporates during the dealloying process, due to the high temperature and the vacuum, leaving apart nanoporous A material	T must be increased to increase the dealloying rate. T must be below the melting point of the precursor	It is related to the time of dealloying. By increasing the process time, the dealloyed volume is increased. The whole volume of the material can be dealloyed

**Table 2:** Summary of the alloys and dealloying techniques reported in this review paper with the key findings.

	Alloy Type	Dealloying Technique and Media	Key Findings	Refs.
Crystalline Alloy	Ti6Al4V	LMD: Mg	low Al on the surface, Studied effect of crucible material on LMD	[78]
	Ti6Al7Nb	LMD: Mg	Tunable pore size, pore shape, and pore depth, reduced Al conc. by 48% on the surface, improved cytocompatibility and bone formation	[79]
	Ti_x_Zr _(100-x)y_Cu_100-y_	LMD: Mg	Tunable surface functionality such as, stiffness, strength, Young’s modulus, cytocompatibile	[82]
	Ti_20_Hf_20_Cu_60_, Ti_25_Hf_10_Cu_60_, Ti_30_Hf_10_Cu_60_	LMD: Mg	Developed metal-polymer composites mimicking mechanical behaviour of cortical bone	[83]
	Ti_27.2_Nb_3_Cu_69.8_ and Ti_29.2_Fe_3.9_Cu_66.9_	LMD: Mg	Tunable phase formation, microstructure, high yield strength and low Young’s modulus	[84]
	Ti_47.5_Mo_2.5_Cu_50_	LMD: Mg	Dendritic microstructure, Elastic modulus near human cortical bone	[85]
	(TiZrCr)_20_Cu_80_	LMD: Mg	Dealloyed multicomponent alloy	[86]
	Ti_50_Sc_50_	CD: HNO_3_	np-Ti by spinodal decomposition	[87]
	(TiMo)_30_Cu_70_, Ti_30_Cu_70_	SSD: Mg	hierarchical structure with improved mechanical properties and bioactive surfaces	[88]
	Ti_20_Cu_80_, Ti_30_Cu_70_, Ti_40_Cu_60_, Ti_15_Zr_15_Cu_70_, and Ti_22.3_Nb_7.7_Cu_70_	LMD: Mg	Np-metal composite with bisphenol F epoxy polymer mimicking the elastic behaviour of human bones	[89]
	Ti_50_Cu_50_	LMD: Mg	Ti-Mg nanocomposites with low Young’s modulus and moderate yield strength	[90]
	Ti_30_Cu_70_	SSD: Mg	suggested pore-forming mechanism is a solid-state interdiffusion process, found that elastic modulus in np-α-Ti foam follow linear decay fit with increasing ligament size	[92]
	Ti_25_Nb_15_Cu_60_	CD: Nitric Alcohol Solution	Np-structure with 24 GPa elastic modulus	[93]
	Ti_3_Zr_2_Sn_3_Mo_25_Nb	ECD: HF + HNO_3_	Dealloying of as-solution treated TLM alloy created a simple np-topography, while in as-cold rolled TLM alloy created a hierarchical micro/np-topography, cytocompatibile surfaces	[94]
	Ti6Al4V	ECD: NaOH	np-surface with low Al conc., bioactive and cytocompatibile surface with high osteogenic activity	[95]
Bulk Metallic Glass	Ti_30_Cu_70_, Ti_40_Cu_60_ and Ti_50_Cu_50_	ECD: HNO_3_	np-Ti oxide layer with mean diameter of pores 50 nm and the thickness of pore walls 100 nm	[98]
	Y_56_Al_24_Co_20_, Ti_56_Al_24_Co_20_, and Y_20_Ti_36_Al_24_Co_20_	ECD: HNO_3_	np-interconnected microstructure with amorphous phase	[99]
	Ti_45_Y_11_Al_24_Co_20_	CD: H_2_SO_4_	np- Ti oxide surface with improved passivation behaviour in simulated body fluid	[100]
	Ti_30_Cu_70_	LMD: Mg	Hierarchical structure	[101]
	Ti_40_Cu_40_Zr_11_Fe_3_Sn_3_Ag_3_	PSD: NH_4_OH and H_2_O_2_	np-Ti oxide with hydrophilic and hemocompatible surfaces, favourable cytocompatibility and antibacterial activity	[102,103]
	Ti_40_Cu_40_Zr_11_Fe_3_Sn_3_Ag_3_	PSD: HNO_3_	np-Ti oxide with cytocompatible and hemocompatible surface	[104]
	Ti_40_Zr_10_Cu_36_Pd_14_	PSD: HNO_3_	Pd enriched np-Ti oxide surface with enhanced corrosion resistance and biocompatibility.	[105]
	Ti-Cu-(Pd-)	PSD: HNO_3_	evaluation of pitting corrosion and development of corrosion resistance strategies	[106]
	Ti_47_Cu_38_Fe_2.5_Zr_7.5_Sn_2_Si_1_Ag_2_	PSD: HNO_3_	improved corrosion resistance and biocompatibility	[107]
Shape Memory Alloy	NiTi	CD: nitro dioctyl phthalate + H_2_O_2_ + HCl + H_2_SO_4_	Ni depleted np-surface with thickness 130 nm, cytocompatible with dermal mesenchymal stem cells	[111]
	NiTi	CD: FeCl_3_	nanogrid structure, bioactive surface promoting hydroxyapatite growth after 14-day immersion in Hank’s solution	[112]
	NiTi	ECD: HNO_3_	hierarchically porous composite of NiTi/hydrogels with high water retention capacity for antibiofouling performance	[113]

## Data Availability

No new data were created or analyzed in this study. Data sharing is not applicable to this article.
